# Re-analysis of protein data reveals the germination pathway and up accumulation mechanism of cell wall hydrolases during the radicle protrusion step of seed germination in *Podophyllum hexandrum*- a high altitude plant

**DOI:** 10.3389/fpls.2015.00874

**Published:** 2015-10-26

**Authors:** Vivek Dogra, Ganesh Bagler, Yelam Sreenivasulu

**Affiliations:** ^1^Biotechnology Division, Council of Scientific and Industrial Research-Institute of Himalayan Bioresource TechnologyPalampur, India; ^2^Centre for Biologically Inspired System Science, Indian Institute of Technology JodhpurJodhpur, India

**Keywords:** protein-protein interactions, network modeling, *Podophyllum* seed germination protein interaction network (PGN), radicle protrusion, seed germination proteins, cell wall hydrolases

## Abstract

*Podophyllum hexandrum* Royle is an important high-altitude plant of Himalayas with immense medicinal value. Earlier, it was reported that the cell wall hydrolases were up accumulated during radicle protrusion step of *Podophyllum* seed germination. In the present study, *Podophyllum* seed Germination protein interaction Network (PGN) was constructed by using the differentially accumulated protein (DAP) data set of *Podophyllum* during the radicle protrusion step of seed germination, with reference to *Arabidopsis* protein–protein interaction network (*At*PIN). The developed PGN is comprised of a giant cluster with 1028 proteins having 10,519 interactions and a few small clusters with relevant gene ontological signatures. In this analysis, a germination pathway related cluster which is also central to the topology and information dynamics of PGN was obtained with a set of 60 key proteins. Among these, eight proteins which are known to be involved in signaling, metabolism, protein modification, cell wall modification, and cell cycle regulation processes were found commonly highlighted in both the proteomic and interactome analysis. The systems-level analysis of PGN identified the key proteins involved in radicle protrusion step of seed germination in *Podophyllum*.

## Introduction

*Podophyllum hexandrum* Royle (= *SinoPodophyllum hexandrum*) also known as “Indian mayapple,” is an important Himalayan medicinal herb exploited for its etoposides which are potential anticancer compounds. Erratic seed germination and poor seedling establishment have resulted in shrinking of their natural resource base. Being a high altitude plant growing in stringent climatic conditions, as an adaptive mechanism, the embryo of seed is surrounded by thick walled, multi layered endosperm tissue and thick testa (Sreenivasulu et al., 2009). However, at the same instance these protective layers create a physical barrier to water uptake and provide a constraint against radicle emergence during seed germination (Sreenivasulu et al., 2009; Dogra et al., 2013; Dogra and Sreenivasulu, 2015).

Seed germination starts with uptake of water when kept for imbibition of the dry seed and ends with the radicle emergence (Bewley and Black, 1994). DNA integrity, quality of the mRNAs stored during embryo maturation and proteostasis are the main contributors for a seed to its successful germination (Rajjou et al., 2012). In order for a seed to complete germination, the growth potential of the radicle must overcome the tissue resistance of the seed covering layers (Leubner-Metzger et al., 1995; Bewley, 1997; Rosental et al., 2014). Testa rupture and endosperm rupture are two sequential events during the germination of many species, including *Arabidopsis* and *Podophyllum* (Leubner-Metzger et al., 1995; Krock et al., 2002; Petruzzelli et al., 2003; Liu et al., 2005; Sreenivasulu et al., 2009). Different plant species has distinctly different cell wall architecture at its micropylar endosperm. Weakening of micropylar endosperm during seed germination by cell wall hydrolases is a widely conserved mechanism. In seeds, where the endosperm acts as a mechanical barrier, either endosperm weakening is essential for endosperm rupture or sufficient force to be generated within the embryo axis to physically break through, or both, is needed for radicle protrusion. So far, no key event(s) has been identified that results in its completion of this process (Nonogaki et al., 2010). Nonogaki (2014) emphasized the need for understanding the embryo-endosperm interaction and integrate them to re-draw a potential comprehensive germination scheme. Endosperm weakening is controlled by phytohormone balance (ABA-GA ratio) where GA promotes and ABA inhibits the weakening process. The higher level of GA hormone up regulates the expression of genes/proteins required for decline in mechanical resistance offered by the micropylar endosperm and enhances the growth potential of the embryonic axis (Bewley, 1997; Sreenivasulu and Amritphale, 1999; Finch-Savage and Leubner-Metzger, 2006; Sreenivasulu et al., 2009; Rana and Sreenivasulu, 2013). In our earlier studies on *Podophyllum hexandrum*, it was found that presence of thick-walled cells at the micropylar endosperm, delays the radicle protrusion during seed germination (Sreenivasulu et al., 2009). Proteomic analyses during radicle protrusion step of seed germination in *Podophyllum* (Dogra et al., 2013) revealed up-accumulation of cell wall hydrolases i.e., β-1, 3-glucanase, XET, pectinmethylesterases etc., which might alter the thick cell walls of the micropylar endosperm tissue. Apart from these, a large number of other proteins which are involved in metabolism (carbohydrate and amino acid metabolism), ABA/GA signaling and stress related proteins also differentially accumulated during germination (Dogra et al., 2013). We confirmed that these differentially accumulated proteins (DAPs) are critical for germination processes in *Podophyllum* (Dogra et al., 2013; Dogra and Sreenivasulu, 2015).

In the era of systems biology, the available protein–protein interaction data enable systems level study of protein interaction networks (Mering et al., 2002). The molecular interactome for seed germination in *Arabidopsis* has been explored in an earlier study by Bassel et al. (2011). Interactome studies will help us to understand how these DAPs are interacting with each other in promoting germination process in *Podophyllum*.

In the present study, we constructed protein–protein interaction network which represents the interwoven processes involved in between the DAPs of *Podophyllum* seed during the radicle protrusion step of its germination. Further, proteins central to the interactome and that are critical for germination in *Podophyllum* were also identified by using graph theoretical methodology. The data on the construction and analysis of the *Podophyllum* seed germination protein ineractome during radicle protrusion step are presented in this paper.

## Materials and methods

### Identification of *Podophyllum* germination proteins (PGPs) from proteomics studies

DAPs during the radicle protrusion step of seed germination of *Podophyllum* i.e., PGPs reported by Dogra et al. (2013), are used as the data set for the construction of *Podophyllum* germination protein interaction network i.e., PGN. *Arabidopsis thaliana* orthologs of the PGPs were identified using BLASTP algorithm in The Arabidopsis Information Resource (TAIR BLAST 2.2.8), where the expected cut-off was less than 0.5 (Altschul et al., 1997). The PGPs utilized in the present study was identified using proteome analysis by MALDI-ToF/ToF (MS/MS) and subsequent protein database searches of all plants using MASCOT algorithm. As there is no much information is available on *Podophyllum* genome and also because of the limited proteome information availability in plants, it is difficult to obtain orthologs for all the *Podophyllum* proteins by using higher cut-off values and with other algorithms like RBH. Hence the sequences of PGPs were searched in TAIR BLAST algorithm which employs BLAST and PSI-BLAST scripts, to find out the most similar matches. These were then considered as the best probable orthologs of respective PGPs in *Arabidopsis*. The details of PGPs and their orthologs in *Arabidopsis* are provided in Table S1.

### Construction of *Podophyllum* germination protein interaction network (PGN)

Proteins are the functional macromolecules that are central in regulation of biological processes. We intended to integrate empirical data of PGPs obtained from proteomic studies and those of protein–protein interactions (PPIs), in order to construct a representative interaction network underlying radicle protrusion step of seed germination mechanism, in general and particularly in *Podophyllum hexandrum*. Since no data of PPIs in *Podophyllum* are available we used PPIs of *Arabidopsis* as a reference. It was considered that the orthologs may have similar functions across the species, but it might not be true for all proteins. The germination mechanisms and their specifiers are not same in all plant species, as the seeds of different plant species have their own kind of embryonic and extra embryonic germination barriers. Hence these are the limitations of this approach, which otherwise has a potential applicability.

A protein interactome of *Arabidopsis thaliana* was constructed using the interaction data available from *Arabidopsis thaliana* Protein Interaction Network (*At*PIN) (Brandão et al., 2009). *At*PIN includes all interactions reported so far i.e., the *Arabidopsis thaliana* Protein Interactome Database (*AtPID*) (Cui et al., 2008), the predicted interactome for *Arabidopsis* (Geisler-Lee et al., 2007), the *Arabidopsis* protein–protein interaction data curated from the literature by TAIR curators as found in repositories for interaction datasets as BIOGRID (Stark et al., 2006) and IntAct (Hermjakob et al., 2004). In particular, the *At*PIN represents a comprehensive protein interaction network of *Arabidopsis thaliana*, based on empirical data (Brandão et al., 2009). It constitutes of 15,163 proteins and 96,827 interactions among them. The giant component *At*PIN constitutes of 99.49% of the *At*PIN proteins (Figure S3). In order to construct *Podophyllum* seed germination protein interactome during the radicle protrusion step, the functional neighborhood of PGPs was obtained with the help of *At*PIN data. First, the PGPs were mapped onto the *At*PIN to obtain the functionally equivalent core set of interactions. Out of 88 PGPs, 68 were mapped onto *At*PIN. Proteins that directly interact with each other are known to play roles in similar biochemical processes (Hartwell et al., 1999). Hence, in order to extend the PGP information into the *Podophyllum* seed Germination protein interaction Network (PGN), the direct interactors of PGP orthologs in *At*PIN were used.

### GO enrichment

Gene Ontology (GO) enrichment analysis was used to identify characteristic biological attributes of a gene set. It is based on the hypothesis that functionally related genes accumulate in corresponding GO categories. Gorilla (Eden et al., 2009), a tool to identify enriched GO terms, was used to obtain biological attributes characterizing PGN genes. It uses the hyper geometric distribution to identify enriched GO terms in a given set of genes. GO enrichment was performed using “two unranked lists of genes” mode, with an “unranked target set” in the background of an “unranked source set.” “Significantly enriched GO terms” were identified, for Biological Process and Molecular Function with *p* < 0.001 and those having at most 100 genes associated with, in the source data (B90). The latter criterion is used to weed out terms those are too generic. The genes from the giant component of the PGN (target) were enriched against all genes present in *At*PIN (source). These GO enrichment experiment helped us obtain biological attributes that characterize the genes from the functional neighborhood of the *Podophyllum* germination seed genes, in the background of *At*PIN genes universe.

### Complex network analysis for PGN

The PGN was modeled and analyzed to obtain its graph theoretical parameters. These parameters include those representing topological as well as dynamical features: degree (Barabási and Oltvai, 2004), betweenness (Freeman, 1977; Brandes, 2001), closeness (Freeman, 1978), Radiality (Newman, 2001), clustering coefficient (Barabási and Oltvai, 2004), and topological coefficient (Stelzl et al., 2005). Degree corresponds to the number of nodes adjacent to a given node v, where adjacent means directly connected. Betweenness (normalized) enumerates number of shortest paths from all pairs of vertices passing through the node of interest. The higher the value of stress/betweenness, the higher is the relevance of the protein as a critical mediator of regulatory molecules and/or functional modules. Two additional network metrices (closeness and radiality) embodying dynamical interplay of the protein interactions, were also used to identify proteins that are central to the interactome. The closeness of a node v is the reciprocal of sum of shortest paths between the node v and all other nodes in the graph. The higher the closeness of a node, the closer it is to the center of the network. The radiality of a node v represents average shortest path between the node v and all other nodes in the graph with respect the diameter (δ_G_) of the network. Radiality is a network metric that enumerates the relative centrality of a node.

### Identification of key proteins for *Podophyllum* germination

We intended to identify key proteins of PGN that are central to the germination protein interactome with the help of graph theoretical parameters. A heat map of selected network parameters depicting the Pearson's Correlation coefficients was generated using R packages. Based on the heat map, the following four parameters were adjudged to have best predictive values for the identification of topologically relevant (key) nodes: degree, betweenness, closeness and radiality. Further the PGN nodes were ranked according to each of the chosen parameters. Top50 nodes, for each of the parameters, were identified and unique proteins were selected for the correlation between closeness and radiality and degree and stress. Proteins found to be central to network were searched in protein knowledgebase UniProtKB (http://www.uniprot.org/) for determining their functions. A set of 60 key proteins was thus identified on the basis of their functional relevance to seed germination.

## Results and discussion

### Construction of protein–protein interactome of *Podophyllum* during the radicle protrusion step of seed germination

**Figure 1:**
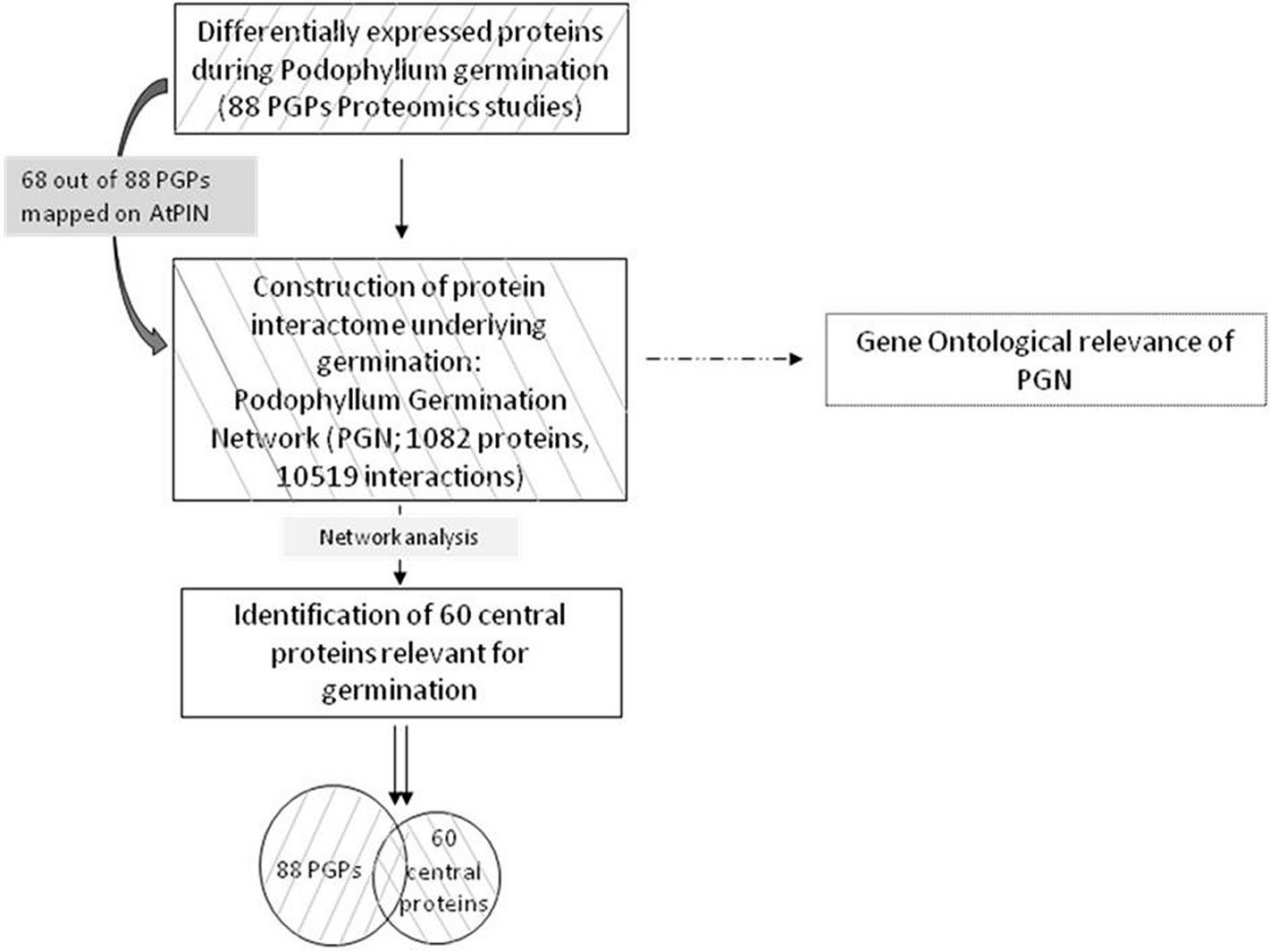
**Schematic outlay of the strategy implementing a systems approach for identification of proteins relevant in ***Podophyllum*** radicle protrusion step of seed germination**. Starting from a seed set of proteins identified with proteomic studies, a protein interactome of underlying molecular mechanisms was constructed, followed by identification of a superset with the help of complex network analysis.

Proteomic analysis of un-germinated and germinating *Podophyllum* seeds identified 88 DAPs (Dogra et al., 2013) that were either up- or down- accumulated (Figure S1, Table S1). In the present study, a possible protein–protein interactome map was constructed by using these DAP data set. The first ortholog interactors of this network represent the possible members of the radicle protrusion step of seed germination mechanism at least in *Podophyllum*. Schematic outlay of the strategy implemented in the present study for understanding the *Podophyllum* radicle protrusion step of seed germination mechanism was shown in Figure 1.

**Figure 2:**
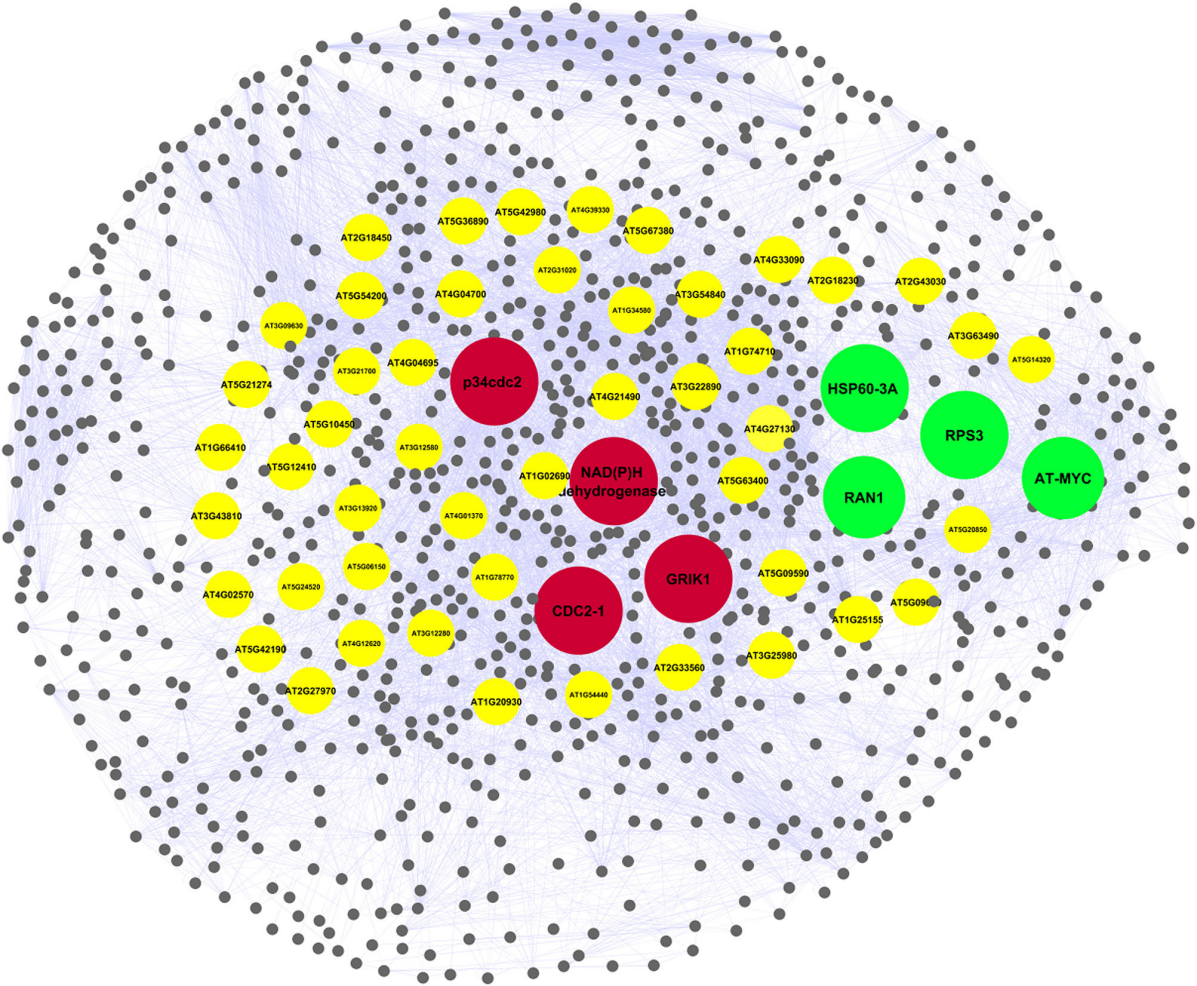
*****Podophyllum*** Germination protein interaction Network (PGN) representing molecular mechanisms underlying ***Podophyllum*** radicle protrusion step of seed germination**. The giant component comprising of 10,466 interactions among 1028 proteins was used for the network analysis. Key proteins identified by network analysis which were relevant for the germination are highlighted in yellow. Proteins overlapped with initial proteomics based dataset are highlighted in red (down-) and green (up-accumulated). The shape of the network was rendered to resemble the seed of *Podophyllum hexandrum*.

**Figure 3:**
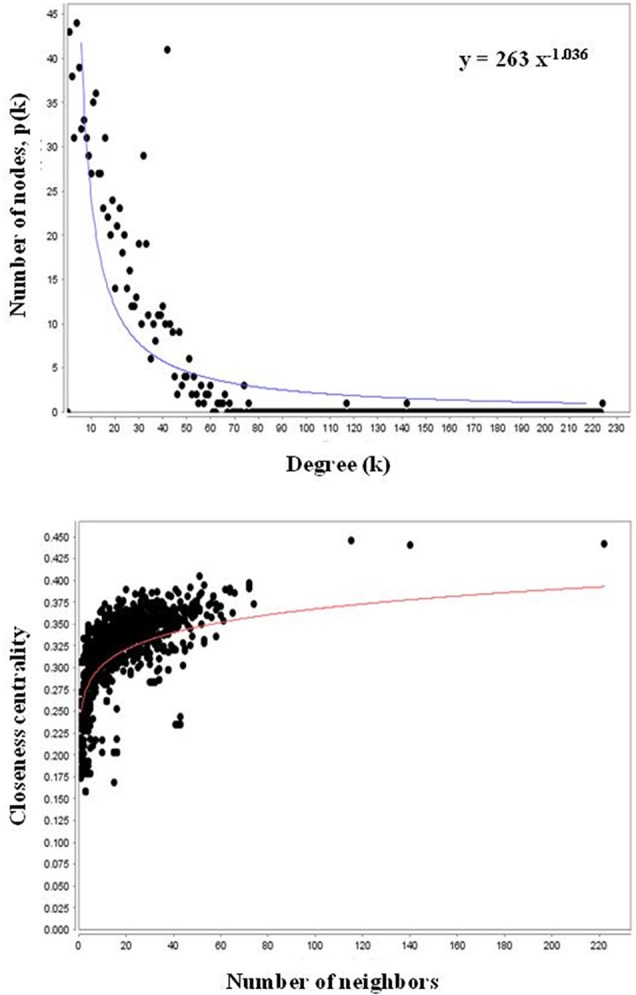
**Scale-free nature of degree and closeness centrality distributions of PGN**. The distributions of degree and closeness centrality show a scale-free nature.

*At*PIN comprises of 96,827 protein interactions among 15,163 proteins (Figure S2 and Table S2). Orthologs is composed of PGPs in *Arabidopsis thaliana* were considered as functional equivalent representatives and were mapped onto the *At*PIN. Out of 97 PGPs, 68 proteins with 17 interactions among them were mapped successfully to obtain a core network of *Podophyllum* radicle protrusion step of seed germination. The core network obtained was not extensive and detailed enough to represent intricate mechanisms that are known to characterize seed germination. Hence, we aimed to obtain a more detailed protein interactome with the help of systems-level data of plant processes that are available. Proteins that interact with each other are known to be associated with similar processes and functions (Hartwell et al., 1999; Barabási and Oltvai, 2004; Randhawa and Bagler, 2012; Vashisht and Bagler, 2012). The constructed PGN constitutes of 10,519 interactions among 1082 proteins including experimentally identified PGPs as their direct interactors. It is comprised of a giant cluster (1028 proteins) and a few small clusters having only 1–6 nodes with no or very few interactions (Figure S3). The network analysis of the fragmented clusters could not be performed as many of the parameters such as betweenness are not computable if it considered as a complete network (along with the small clusters) instead of only the giant component. We excluded these small clusters from the analysis because the proteins of these clusters do not have any functional relevance toward germination and also not having any reported interactions. Probably, these proteins might have yet unknown interactions which might be important for seed germination mechanism. The giant cluster of PGN having 10,466 interactions (Table S2) was used for further downstream analysis (Figure 2). It was observed that PGN is characterized by a scale-free degree distribution, as reported for other molecular interaction networks (Randhawa and Bagler, 2012; Vashisht and Bagler, 2012), indicating presence of hubs with exceptional number of interactions (Figure 3). Owing to their role in the topology and information flow across the PGN interactome, few key proteins seem to play a key role in emerging dynamics of germination mechanism.

### Gene ontological relevance of PGN

**Figure 4:**
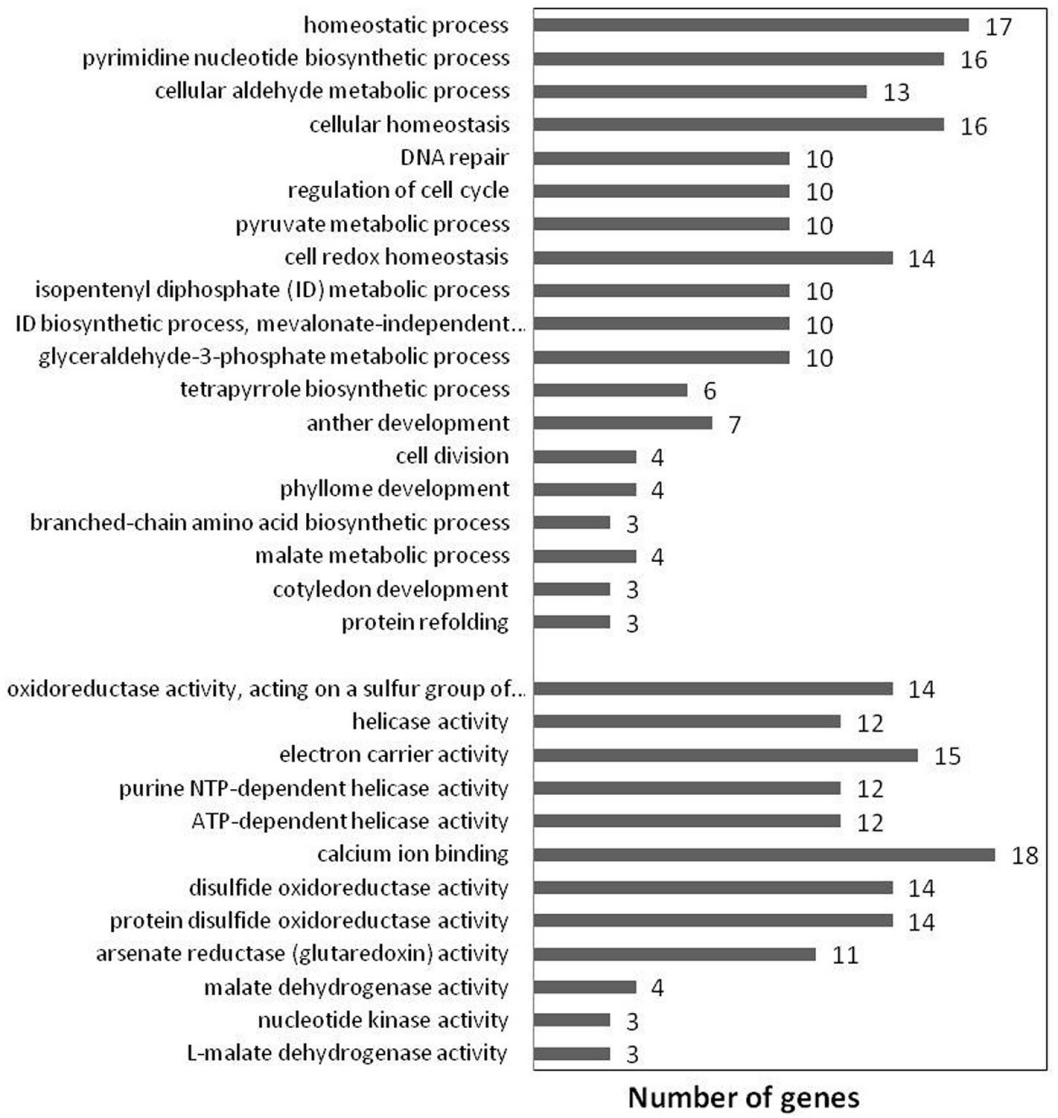
**Significantly overrepresented GO terms for ***Podophyllum*** Germination Network, reflecting processes and functions known to be relevant for seed germination**.

The functional relevance of the PGN gene-set for the ontological correlates was evaluated by conducting GO enrichment analysis in the background of *At*PIN gene-set with the help of Gorilla (Eden et al., 2009). Significantly enriched Molecular Function (MF) and Biological Processes (BP) GO terms that are specific to PGN (*B* ≤ 100) were obtained after rejecting terms that are highly generic (*B* > 100). These significantly enriched terms represent GO categories that are overrepresented in PGN and are ontological descriptors of its functions and processes. We obtained 32 (12 for MF and 20 for BP) such significantly enriched GO terms that characterize the network (Figure 4). Germination is a complex process during which the imbibed mature seed quickly shifts from a maturity- to a germination-driven program of development and prepares for seedling growth (Bewley and Black, 1994; Finch-Savage and Leubner-Metzger, 2006; Nonogaki, 2006). GO enrichment analysis of PGN indeed deduced a mechanism which is more relevant for seed germination. Few of the key processes involved in germination are response to cellular damage, cell cycle regulation, homeostasis, *de novo* mRNA and protein synthesis (Bewley, 1997).

Dehydration and rehydration during seed development and germination are associated with high levels of oxidative stress, resulting in damage to membranes, cellular compartments, proteins and DNA (Powell and Matthews, 1978). The seed activates a number of repair mechanisms during imbibition to respond DNA and protein damage due to cumulative effects of temperature, moisture, oxygen and ROS levels during seed maturation, after-ripening and storage. Ventura et al. (2012) emphasized the need to understand the complexity of the networks of DNA damage control/repair functions in plants. The initiation of germination process depends on cell cycle regulation mechanisms, especially G1 check point control, to cope up with DNA damage and to ensure the maintenance of chromosomal integrity (Vazquez-Ramos and Sanchez, 2003). DNA and protein repair machinery also needs to be activated which determines vigor and the longevity of the seed ultimately marks the phenotype of germination process (Ogé et al., 2008; Ventura et al., 2012; Verma et al., 2013). Verma et al. (2013) identified a change in the content of L-isoaspartyl methyltransferase1 protein during seed germination in chickpea, which is also associated with enhancement of seed vigor and longevity. Mobilization of seed storage proteins is considered as a post-germinative event occurring during seedling establishment (Bewley, 1997; Penfield et al., 2005). However, Gallardo et al. (2001) documented that this mobilization can be initiated during the imbibition phase of *Arabidopsis* seed germination. The seed, in response to extensive changes in redox state during germination, works toward redox homeostasis through thioredoxins, protein disulfide oxidoreductases and glutaredoxins. Malate dehydrogenase plays important role by participating in lipids mobilization and by maintaining redox homeostasis. Expression of nucleic acid helicases, such as ATP dependent DEAD box RNA helicase, increases to maintain viability of seeds and for synthesis of new enzymes to sustain growth and development of the seedlings (Li et al., 2001). Increased nucleotide kinase activity helps to maintain stable NTP levels through nucleotide homeostasis in various metabolic pathways such as protein and DNA synthesis and signal transduction (Yano et al., 1995). Calcium, which plays a pivotal role in plant responses to several stimuli, is known to promote germination by inducing the calcium binding proteins which regulate ABA/GA signaling as well as by assisting in reserve mobilization (Vandana and Bhatla, 2009).

### Analysis of PGN identified key proteins of radicle protrusion step of seed germination

**Figure 5:**
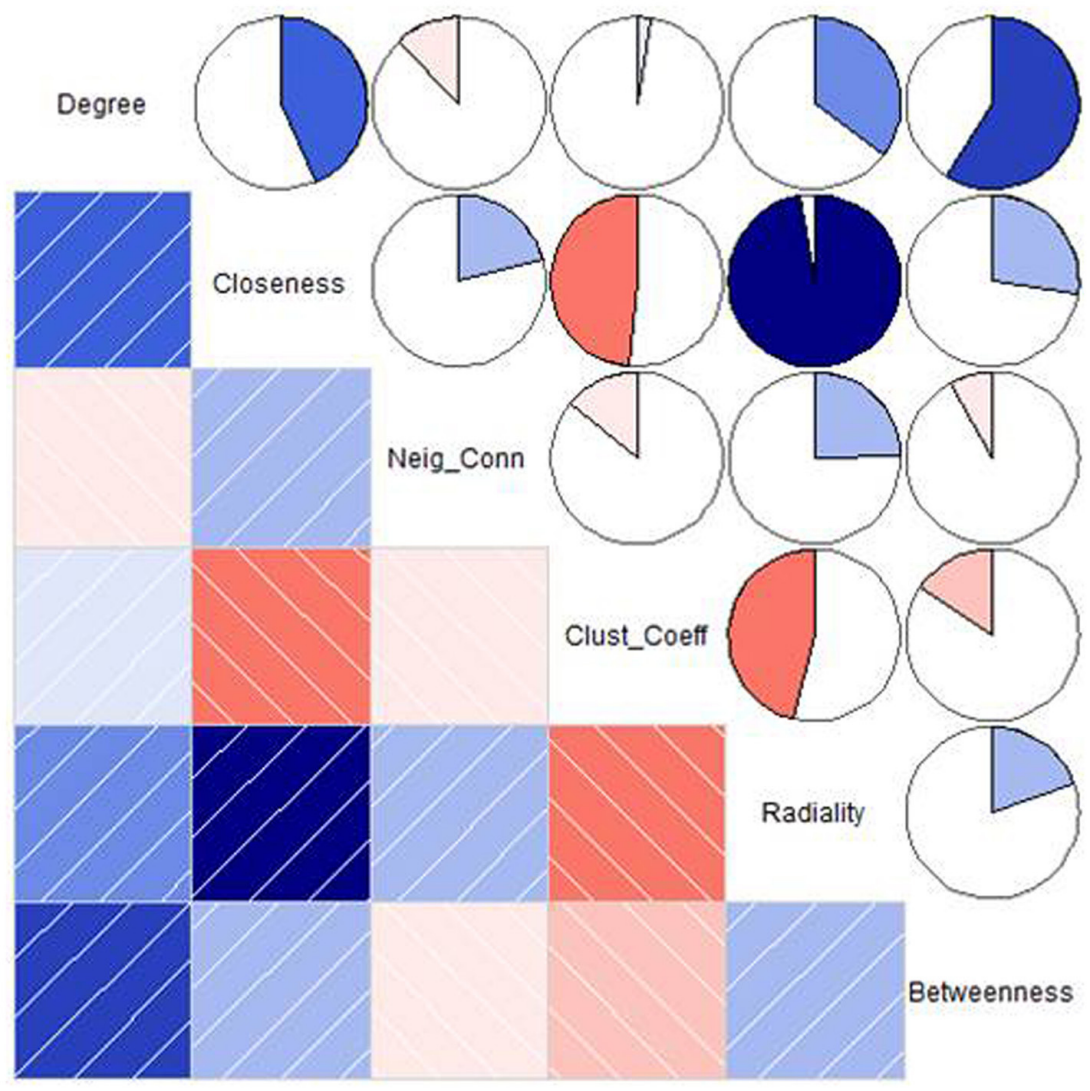
**A correlogram depicting the Pearson's correlation coefficients between the network parameters**. In the lower triangle the correlations are represented as a color-coded shaded chart indicating positive and negative correlations. The upper triangle depicts pie chart. Shades of blue indicating positive correlations (the darker shade the stronger is the positive correlation) and shades of red indicating negative correlations (the darker shade the stronger is the negative correlation).

**Figure 6:**
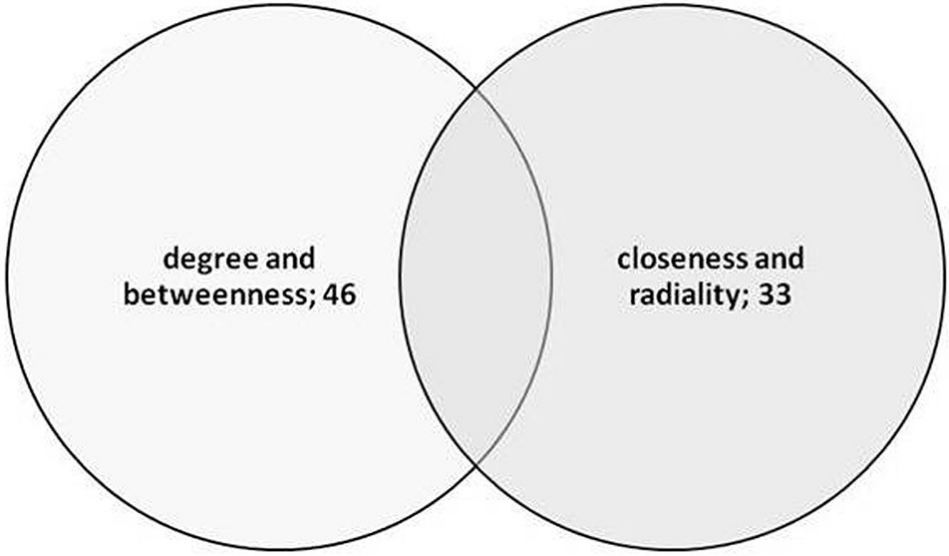
**Venn diagram showing the overlapping of top 50 nodes in correlated combinations of network attributes degree, betweenness and closeness, radiality**.

The main aim of the study was to identify “key proteins” that are associated with the germination mechanism and central to the network which provide the structural stability and having information dynamics of the PGN. To assess this, six network parameters i.e., degree, closeness centrality, neighborhood connectivity, clustering coefficient, radiality, and betweenness centrality were used which reflect the critical importance of the proteins central to the PGN. These parameters reflect the global as well as local role of the central proteins (nodes) in the interactome as topological hubs and bottlenecks for the information flow. The heat map in Figure 5 depicts pair wise correlations among these network metrics. Based on the mutual correlations profile, we chose the following two pairs of network metrics for identification of central proteins that reflect their role in the PGN: (a) degree and betweenness and (b) closeness and radialilty. Thus, among the central proteins we were able to identify richly interacting protein hubs and those mediating exceptional number of pair wise interactions across the interactome. These central proteins, thus identified, also include proteins those are of potential regulatory relevance due to their placement in the PGN. For each of the parameters, nodes with Top50 values were identified. A total of 57 proteins were obtained for the degree (nodes of Top50), whereas 50 nodes each were obtained for the remaining three parameters i.e., closeness, radiality, and stress (Table S3). A list of 74 and 50 “unique” central proteins were identified from these parameter pairs, respectively. This list of 124 central proteins was further pruned on the basis of the functional relevance to seed germination, which resulted in 60 “key proteins.” The functional relevance of these central proteins was ascertained on the basis of GO annotations from The *Arabidopsis* Information Resource (TAIR, http://www.arabidopsis.org). Among the key proteins, 46 were uniquely obtained from the degree and betweenness based central proteins; 33 from those based on closeness and radiality. Nineteen of these key proteins were found to be common to both the metric pairs list (Figure 6).

**Figure 7:**
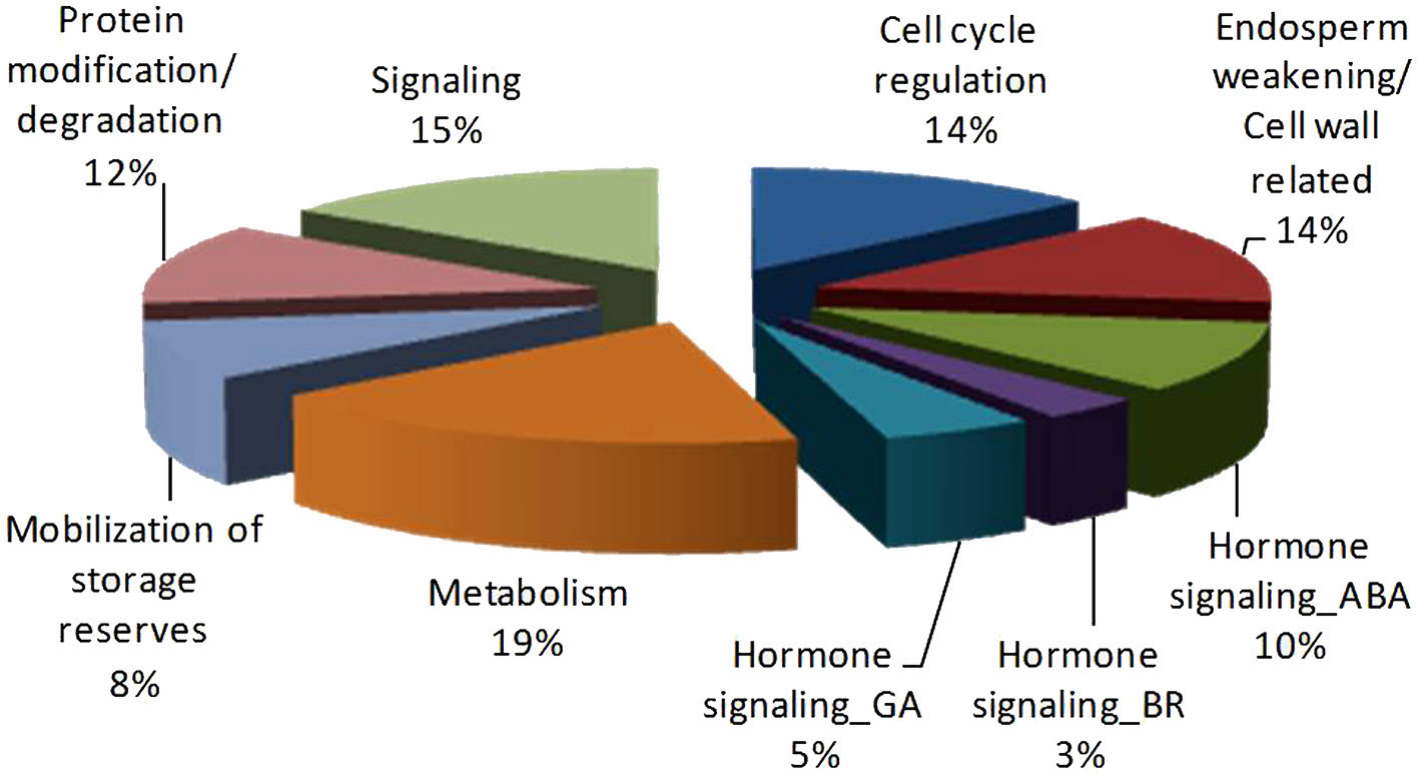
**Pie-chart representing germination relevant Key proteins identified from ***Podophyllum*** Germination interactome analysis**. Functional categorization of 60 central proteins of PGN.

The key genes thus identified from the network analysis are mainly involved in germination relevant processes such as cell cycle regulation, cell wall metabolism/endosperm weakening, hormonal signaling and/or metabolism (ABA, BR, and GA), transportation, protein folding, modification and ubiquitination, metabolism including transcription and translation regulation, protein synthesis, transportation and mobilization of storage reserves (Figure 7 and Table 1).

**Table 1:** **Central proteins identified from *Podophyllum* germination network, critically important for radicle protrusion step of seed germination mechanism**.

**S. no**.	**Locus id**	**GO term**	**Protein name**
**CELL CYCLE REGULATION**			
1.	AT3G48750	GO:0009793	Cyclin-dependent kinase A-1
2.	AT2G27970	GO:0007049	CDK-subunit 2 (CKS2)
3.	AT1G20930	GO:0004693	Cyclin-dependent kinase B2 (CDKB2)
4.	AT3G25980	GO:0007094	Mitotic arrest-deficient 2 (MAD2)
5.	AT2G33560	GO:0007094	Budding uninhibited by benzymidazol 1-related (BUBR1)
6.	AT5G20850	GO:0006355	RAS associated with diabetes protein 51 (RAD51)
7.	AT4G12620	GO:0009567	Origin of replication complex 1B (ORC1B)
8.	AT3G12280	GO:0000082	Retinoblastoma-related protein 1 (RBR1)
9.	AT3G54180	GO:0004672	Cyclin-dependent kinase B1;1 (CDKB1;1)
10.	AT4G35620	GO:0051726	Cyclin B2;2 (CYCB2;2)
11.	AT5G06150	GO:0000079	Cyclin B1;2 (CYCB1;2)
**ENDOSPERM WEAKENING/CELL WALL RELATED**			
12.	AT5G36890	GO:0004553	Beta-glucosidase 42 (BGLU42)
13.	AT3G18660	GO:0080116	Glucuronic acid substitution of xylan 1 (GUX1)
14.	AT5G24520	GO:0009963	Transparent testa glabra 1 (TTG1)
15.	AT1G63650	GO:0009909	Enhancer of glabra 3 (EGL3)
16.	AT4G39330	GO:0009809	Cinnamyl alcohol dehydrogenase 9 (CAD9)
17.	AT1G04750	GO:0016192	Vesicle-associated membrane protein 721 (VAMP721)
**HORMONE SIGNALING (ABA/GA/SA/AUXIN/ETHYLENE)**			
18.	AT2G31020	GO:0008202	Oxysterol binding-related protein 1A (ORP1A)
19.	AT5G67380	GO:0004672	Casein kinase II (CK2) catalytic subunit (alpha 1)
20.	AT4G01370	GO:0009738	MAP kinase 4 (MPK4)
21.	AT1G71860	GO:0000079	Protein tyrosine phosphatase 1 (PTP1)
22.	AT5G10450	GO:0005515	14-3-3 Protein G-box factor14 lambda (14-3-3LAMBDA)
23.	AT5G12410	GO:0009560	THUMP domain-containing protein
24.	AT1G74710	GO:0000165	Isochorismate synthase 1 (ICS1)
**METABOLISM**			
***General metabolism***			
25.	AT1G25155	GO:0008152	Anthranilate synthase beta subunit
26.	AT4G21490	GO:0003954	NAD(P)H dehydrogenase B3 (NDB3)
27.	AT1G07180	GO:0003954	Internal NAD(P)H dehydrogenase in mitochondria
28.	AT3G22890	GO:0004781	ATP sulfurylase 1 (APS1)
***Metabolism related to protein translation***			
29.	ATCG00800	GO:0000312	Plastidial ribosomal protein S3
30.	AT3G63490	GO:0003723	Plastidial ribosomal protein L1 (PRPL1)
31.	AT5G14320	GO:0019288	Plastidial ribosomal protein S13/S18 family
32.	AT2G43030	GO:0019288	Ribosomal protein L3 family protein
33.	AT3G09630	GO:0042545	Ribosomal protein L4/L1 family
34.	AT3G13920	GO:0008026	Eukaryotic translation initiation 4A1 (EIF4A1)
35.	AT5G35910	GO:0009560	Polynucleotidyl transferase
***Mobilization of storage reserves***			
36.	AT2G18230	GO:0000287	Pyrophosphorylase 2 (PPA2)
37.	AT2G18450	GO:0000104	Succinate dehydrogenase 1-2 (SDH1-2)
38.	AT4G33090	GO:0004177	Aminopeptidase M1 (APM1)
39.	AT5G09660	GO:0005975	Peroxisomal NAD-malate dehydrogenase 2 (PMDH2)
**PROTEIN MODIFICATION, UBIQUITINATION, AND STRESS DETOXIFICATION RELATED PROTEINS**			
40.	AT3G12580	GO:0006457	Heat shock protein 70 (HSP70)
41.	AT3G13860	GO:0042026	Heat shock protein 60-3A (HSP60-3A)
42.	AT5G09590	GO:0051082	Heat shock cognate 70-5 (HSC70-5)
43.	AT5G52640	GO:0005618	Heat shock protein 90-1 (HSP90-1)
44.	AT1G78770	GO:0000087	Anaphase promoting complex 6 (APC6)
45.	AT5G42190	GO:0004842	Arabidopsis SKP-like 2 (SKP1B)
46.	AT4G02570	GO:0000151	CULLIN 1 (CUL 1)
47.	AT5G42980	GO:0045454	Thioredoxin 3 (TRX3)
**SIGNALING AND TRASNPORTATION**			
48.	AT4G04700	GO:0005509	Calcium-dependent protein kinase 27 (CPK27)
49.	AT4G04695	GO:0004683	Calcium-dependent protein kinase 31 (CPK31)
50.	AT1G66410	GO:0005509	Calmodulin 4 (CAM4)
51.	AT3G43810	GO:0019722	Calmodulin 7 (CAM7)
52.	AT5G21274	GO:0005509	Calmodulin 6 (CAM6)
53.	AT3G45240	GO:0004672	Geminivirus REP interacting kinase 1 (GRIK1)
54.	AT5G54200	GO:0007165	Transducin/WD40 repeat-like superfamily protein
55.	AT5G63400	GO:0004017	Adenylate kinase 1 (ADK1)
56.	AT1G34580	GO:0005351	Sugar transport protein 5
57.	AT3G06720	GO:0006886	Importin alpha isoform 1 (IMPA1)
58.	AT1G02690	GO:0000226	Importin alpha isoform 6 (IMPA6)
59.	AT3G54840	GO:0003924	Ras-related small GTP-binding family protein (RABF1)
60.	AT5G20010	GO:0007165	RAS-related nuclear protein-1 (RAN1)

### Analysis of PGN hubs confirmed their involvement in radicle protrusion step of seed germination

#### Cell cycle regulation

In PGN hubs of different gene groups were formed on the basis of their function e.g., cell cycle regulation, cell wall alteration related genes, hormone, cell signaling and metabolism, transportation, protein folding and modification. The initiation of germination process depends on cell elongation and cell division in the cells of the embryonic axis. During cell division a tight regulation is necessary to ensure the maintenance of chromosomal integrity (Vazquez-Ramos and Sanchez, 2003). The cell cycle is regulated at two checkpoints (at least the G1- to S-phase transition and entry into mitosis) through a particular class of protein kinases activity (Pines, 1993). GA regulates cell division and elongation through cyclin B1 (*At5g06150*) and cyclin B2 (*At4g35620*) which are involved in the regulation of cyclin dependent protein kinases (Ogawa et al., 2003). In *Podophyllum* also change in cyclin-dependent kinase A2 was documented during radicle emergence step of seed germination (Dogra et al., 2013). These kinases play a significant role in ensuring passage of cell cycle from G1 to S phase and therefore maintain genomic integrity (Imajuku et al., 2001; Day et al., 2008).

Up regulation of genes specific to G1 to S phase of the cell cycle was reported during GA induced seed germination in *Arabidopsis* by Ogawa et al. (2003). GA regulates cell division and elongation through cyclin dependent protein kinases i.e., cyclin B1 (*At5g06150*) and cyclin B2 (*At4g35620*) which are involved in the regulation of cell cycle (Ogawa et al., 2003). Cyclin-dependent kinase B1 (CDKB1-*At3g54180*) regulates the hypocotyl cell elongation and cotyledon cell development also (Boudolf et al., 2004). Similarly, change in cyclin-dependent kinases was observed in *Podophyllum* during the radicle protrusion step of seed germination (Dogra et al., 2013). In addition to different kinases, a number of other proteins like MAD (*At3g25980*), BuBR1 (*At2g33560*), RAD51, ORC1, and RBR1 were also connected with this functional hub in the PGN. MAD protein function has been reported to regulate the root growth in *Arabidopsis* by Ding et al. (2012) *via* the localization of the kinetochore during cell division. RAS associated with diabetes protein 51(Rad51- *At5g20850*) has role in chromosomal pairing during cell division, and is needed for both meiotic and mitotic recombination during cell division (Ines et al., 2012). Origin of replication complex 1B (ORC1-*At4g12620*), of which the large subunit of the origin recognition complex is involved in defining origins of DNA replication and acts as a transcriptional activator of a subset of genes during various developmental processes (Guo et al., 2009). Retinoblastoma-related protein 1 (RBR) is required for the repression of sucrose inducible embryonic and seed maturation genes after germination; thus RBR connects cell fate switch in seedlings after germination with cell cycle control and also is required for the seedlings to become autotrophic, during seedling establishment (Gutzat et al., 2011). Different proteins of this hub have been involved at various stages of cell cycle (Ogawa et al., 2003; Boudolf et al., 2004). Grouping of these proteins in to a hub in the network confirms that they might be involved in the germination process of both *Arabidopsis* and *Podophyllum* seeds.

### Cell wall metabolism and endosperm weakening

Weakening of the cell walls of the endosperm, especially at the micropylar region, is pre-requisite for germination which will be achieved by the induction of cell wall hydrolases. As a result, radicle protrusion may take place upon concomitant weakening of cell walls of the surrounding micropylar endosperm tissue, thereby decreasing the force required by the radicle to penetrate them. Up accumulation in the content of cell wall hydrolases during germination of different plant species seed has been documented by different researchers e.g., endo-b-mannanase in white spruce (Downie et al., 1997), xyloglucan endotransglycosylase in tomato (Chen et al., 2002) and β-1, 3-glucanase in tobacco (Leubner-Metzger et al., 1996). Besides, up accumulation of expansins in Datura (Mella et al., 1995) and pectin esterases in yellow cedar seeds (Ren and Kermode, 2000) were also reported. In their earlier study of *Podophyllum* radicle protrusion step of seed germination by proteomic analysis, Dogra et al. (2013) characterized the accumulation of cell wall hydrolases such as *Ph*glucanase and xyloglucon endotransglycosylase (*Ph*XET) which alters the micropylar endosperm tissue. Other cell wall remodeling proteins such as expansins and pectin esterases were also found up regulated during the radicle protrusion step of seed germination. In the present study, analysis of PGN identified a hub having involvement of cell wall hydrolysing and cell wall modifying proteins such as beta-glucosidase 42 (BGLU42- *At5g36890*) and glucuronic acid substitution of xylan 1 (GUX1-*At3g18660*). The role of these proteins in seed germination was reported by Lee et al. (2012). Plant cytokinesis is characterized by deposition of cell wall material at the cell division plane, required for the generation of new cell walls. Involvement of VAMP721 and VAMP722 proteins in cell plate formation during the plant cytokinesis was reported (Zhang et al., 2011). Analysis of this hub confirmed that the cell wall hydrolases of this hub are involved in weakening of the extra-embryonic envelopes such as perisperm and endosperm layers. Grouping of these proteins into a hub in the network confirms that they might have a significant role in the germination process of both *Arabidopsis* and *Podophyllum* seeds.

### Hormone signaling and metabolism

Abscisic acid (ABA) and gibberellic acid (GA) along with brassinosteroids (BR) are important phytohormones which regulate various cellular processes in plants, including seed germination. GA and ABA act antagonistically to control seed development and germination (Debeaujon et al., 2000). Increased GA levels negatively regulate ABA levels and, thus, promote seed germination. It was proposed that imbibition induced GA and subsequent initiation of a signaling cascade that leads to synthesis of α-amylases and other hydrolytic enzymes in rice (He and Yang, 2013).

MAPks, serine/threonine-protein kinases, protein kinase phosphatases, protein tyrosine kinases and protein tyrosine phosphatases are important molecules involved in ABA signaling. Mitogen-activated protein kinase MAPK4 (*At4g01370*), casein kinase alpha 1 (CKA1-*At5g67380*), a serine/threonine-protein kinase are known to play important roles in ABA and GA regulation (Brock et al., 2010; Wang et al., 2011). Protein tyrosine phosphatise 1 (PTP1- *At1g71860*) is known to be involved in ABA-dependent processes in *Arabidopsis* seeds (Ghelis et al., 2008). Oxysterol binding protein-related protein 1A (ORP1A-*At2g31020*) is involved in the transportation of lipids especially sterols which are involved in the ABA dependent receptor kinase mediated signaling during seed and pollen germination (Skirpan et al., 2006). Salicylic acid (SA) inhibits the seed germination under normal conditions (Rajjou et al., 2006), but it is also known to promote seed germination by modulating antioxidant activity in *Arabidopsis* under abiotic stress conditions including salinity (Rajjou et al., 2006; Lee et al., 2010). Under high salinity, GA stimulates the SA biosynthesis by inducing the expression of isochorismate synthase 1 (ICS1-*At1g74710*) and alternatively, SA also induces genes encoding GA biosynthetic enzymes, which plays a significant role in GA dependent seed germination process (Garcion et al., 2008). Proteins, such as 50S ribosomal protein L21and 30S ribosomal protein S13, involved in protein synthesis, plastid development and embryogenesis (Yin et al., 2012) are known to be down regulated by ABA during seed germination in *Arabidopsis* (Bassel et al., 2011). Change in the accumulation of 50S ribosomal protein was also observed in *Podophyllum* during the radicle protrusion step of seed germination (Dogra et al., 2013).

Ethylene plays diverse functions, alone or in combinatorial interactions with other hormonal signals in plants. Ethylene is known to promote seed germination by interacting with auxin (Stepanova et al., 2005). It was also showed by these authors that ethylene triggers its signaling by the upregulation of anthranilate synthase α1 & ß1 (*At1g25155*) during seed germination. THUMP domain-containing protein (*At5g12410*) is a transcriptional regulatory protein whose expression is controlled by Auxin levels (Huang et al., 2008). Previously, it has been reported in *Arabidopsis* that the endogenous ethylene plays an essential role for seedling establishment after radicle protrusion through methionine biosynthesis (Gallardo et al., 2002).

### Cell signaling and transportation

Calcium is an essential major plant nutrient required for maintaining cell wall integrity and plasma membrane function, besides its known function in promoting seed germination in some woody plants (Liu et al., 2011). Calcium signaling is mediated through calmodulin, calmodulin dependent kinases (CAMKs) and calcium-dependent protein kinases (CDPKs). These calcium signaling molecules participate in various physiological processes including the accumulation of starch and proteins in immature seeds of rice with response to ABA in *Arabidopsis*. Cytosolic Ca^2+^ concentration also increases in response to GA (Bush, 1996). Thus, calcium signaling proteins such as calmodulins (CAM4–*At1g66410*, CAM6–*At5g21274*, and CAM7–*At3g43810*) and calcium-dependent protein kinases (CPK27–*At4g04700* and CPK31–*At4g04695*) play important roles during seed germination. Change in expression of geminivirus rep interacting kinase 1 (GRIK1–*At3g45240*), a calcium/calmodulin-dependent protein kinase, and plasmamembrane related calcium transporting ATPase 11 was found during *Podophyllum* radicle protrusion step of seed germination, thus supporting their roles in ABA signaling during seed germination (Dogra et al., 2013).

High fluxes of nutrients occur in seeds when storage reserves get accumulated or remobilized during development and germination, respectively. Nutrient movement from the place of synthesis to storage and vice versa involves transport between symplasmically isolated compartments and hence transport across plasma membranes of neighboring cells (Aoki et al., 2006). Ras-related protein RABF1 (*At3g54840*) is a small GTPase superfamily endosomal protein involved in endocytosis (Ueda et al., 2001). GTP-binding nuclear protein Ran-1 (*At5g20010*), another GTPase superfamily protein which is involved in nucleocytoplasmic transport, chromatin condensation and control of cell cycle, was found upregulated during radicle protrusion step of seed germination in *Podophyllum* (Dogra et al., 2013).

### Protein modification, ubiquitination stress detoxification related proteins

Heat shock proteins (HSPs) are molecular chaperones involved in a variety of cellular processes including protein folding, protein transport across membranes, modulation of protein activity, regulation of protein degradation and prevention of irreversible protein aggregation (Su and Li, 2008). Heat shock protein 70 (HSP70–*At3g12580*), heat shock protein 60-3A (HSP60-3A–*At3g13860*) (Dogra et al., 2013) and mitochondrial heat shock cognate (HSC70-5–*At5g09590*), are found to be accumulated during *Podophyllum* radicle protrusion step of seed germination. These proteins might be maintaining and giving stability to the proteins required for germination. Cell wall localized heat shock protein 90-1 (HSP90-1; *At5g52640*) and E3 ubiquitin ligases are the enzymes that confer substrate specificity to the ubiquitin/26S proteasome pathway. SKP1-like protein 1B (*At5g42190*) is involved in ubiquitination and subsequent proteasomal degradation of target proteins. It forms a SCF E3 ubiquitin ligase complex together with cullin-1(CUL1–*At4g02570*), RBX1 and a F-box protein (Shen et al., 2002). This type of regulation in protein degradation contributes significantly to plant development by affecting a wide range of processes such as embryogenesis, hormone signaling, and senescence in plants (Moon et al., 2004).

In response to extensive environmental and physiological changes the seeds undergo redox homeostasis through certain proteins such as thioredoxins and glutredoxins in early phase of seed germination. These proteins are involved in electron carrier activity to maintain their flux during the mobilization of carbohydrates and lipids (Osmundsen et al, 1991; Engel, 1992). Thioredoxin 3 (*At5g42980*) is a crucial member of redox related proteins, whose expression levels are reported to reduce during seed germination in *Medicago truncatula* seeds (Alkhalfioui et al., 2007).

### Metabolism including mobilization of storage reserves and protein synthesis

Seed germination starts with rapid water uptake, followed by start of rapid transcription and translation besides other physiological processes such as mobilization, breakdown of storage reserves and utilization of the broken-down products in growth and expansion of the embryo. Many enzymes involved in mobilization of seed storage reserves, become active upon hydration (Tonguc et al., 2012). In the present PGN, it was identified involvement of a few important enzymes related to the mobilization of storage reserves during *Podophyllum* radicle emergence step of seed germination (Table S3). They are ATP sulfurylase 1 (APS1-*At3g22890*), pyrophosphorylase 2 (PPA2-*At2g18230*), succinate dehydrogenase 1-2 (SDH1-2–*At2g18450*), aminopeptidase M1 (APM1-*At4g33090*), and peroxisomal NAD-malate dehydrogenase 2 (PMDH2–*At5g09660*). *Arabidopsis* homologs such as NAD(P)H dehydrogenase (*At1g07180*) and NAD(P)H dehydrogenase B3 (*At4g21490*) are reported to be expressed in cotyledons, seedlings and roots (Table S3). These are important enzymes belonging to family of oxidoreductases responsible for maintenance of redox state (Elhafez et al., 2006). Expression of NAD(P)H dehydrogenase increased with germination in *Podophyllum hexandrum* (Dogra et al., 2013). Translational machinery proteins such as eukaryotic translation initiation factor 4A1 (*At1g02690*), ribosomal protein L4/L1 family (*At3g09630*), ribosomal protein L3 family protein (*At2g43030*) which are required during seed germination were also identified in the PGN (Table S3). Dry seeds preserve mRNAs stored during their maturation on the mother plant as well as during after-ripening and storage upon release in the environment, which drives the synthesis of proteins required for activating the germination process upon seed imbibition (Rajjou et al., 2004). Polynucleotide transferase or exosome complex exonuclease RRP6 (*At5g35910*) is involved in mRNA turnover and degradation of defective and cryptic transcripts, also in the process of the 3′- extremities of a variety of non-coding RNAs and elimination of RISC cleaved mRNA (Houseley et al., 2007). PGN analysis revealed involvement of many key translational machinery proteins, including the cytosolic, plastidial and mitochondrial protein synthesis related proteins such as eukaryotic translation initiation factor 4A1 (*At1g02690*), ribosomal protein L4/L1 family (*At3g09630*), ribosomal protein L3 family protein (*At2g43030*), which were earlier found to be required during seed germination (Houseley et al., 2007) (Table 2). Translational proteins involved in selective protein synthesis, generally disappear once they play their roles to maintain the homeostasis. For instance, down-accumulation in the content of plastidial ribosomal protein S3 (*Atcg00800*) has been documented in *Podophyllum* during radicle protrusion step of seed germination (Dogra et al., 2013). Various investigations, especially dealing with proteome dynamics during seed germination, revealed the importance of selective stored and nascent mRNAs and their respective encoded proteins to determine the metabolite pool required for the germination completion (He et al., 2011; Sano et al., 2012; Galland et al., 2014). The role of the stored mRNAs during the initial phase of seed germination has also established the epigenetic regulation of seed germination, besides being important for resumption of metabolic activities (Rajjou et al., 2004; Nakabayashi et al., 2005; Kimura and Nambara, 2010; Galland and Rajjou, 2015). Interestingly, stored mRNAs have an inherent regulatory effect over the transcription of new mRNAs (Rajjou et al., 2004).

**Table 2:** **Proteomic expression and functional distribution of 08 regulatory hubs in PGN during *Podophyllum* radicle protrusion step of seed germination**.

**Spot No. (2-DE gel)**	**Protein name**	**Locus Id**	**Expression (Proteome level)**	**Probable function**
7	Internal NAD(P)H dehydrogenase	At1g07180	UP	Metabolism, Redox maintenance
49	Heat shock protein 60-3A (HSP60-3A)	At3g13860	UP	Protein modification/degradation
56	RAS-related nuclear protein (RAN1)	At5g20010	UP	Signaling
59	Enhancer of glabra 3 (AT-MYC)	At1g63650	UP	Cell wall modification related
17	Protein kinase p34cdc2/ Cyclin-dependent kinase A-1	At3g48750	DN	Cell cycle regulation
45	GEMINIVIRUS REP INTERACTING KINASE 1(GRIK1)	At3g45240	DN	Signaling
81	30S ribosomal protein S3	AtCg00800	DN	Metabolism- translation
87	Cell division control protein 2 homolog 1	At3g54180	DN	BR signaling

### Regulatory roles of key proteins of PGN

**Figure 8:**
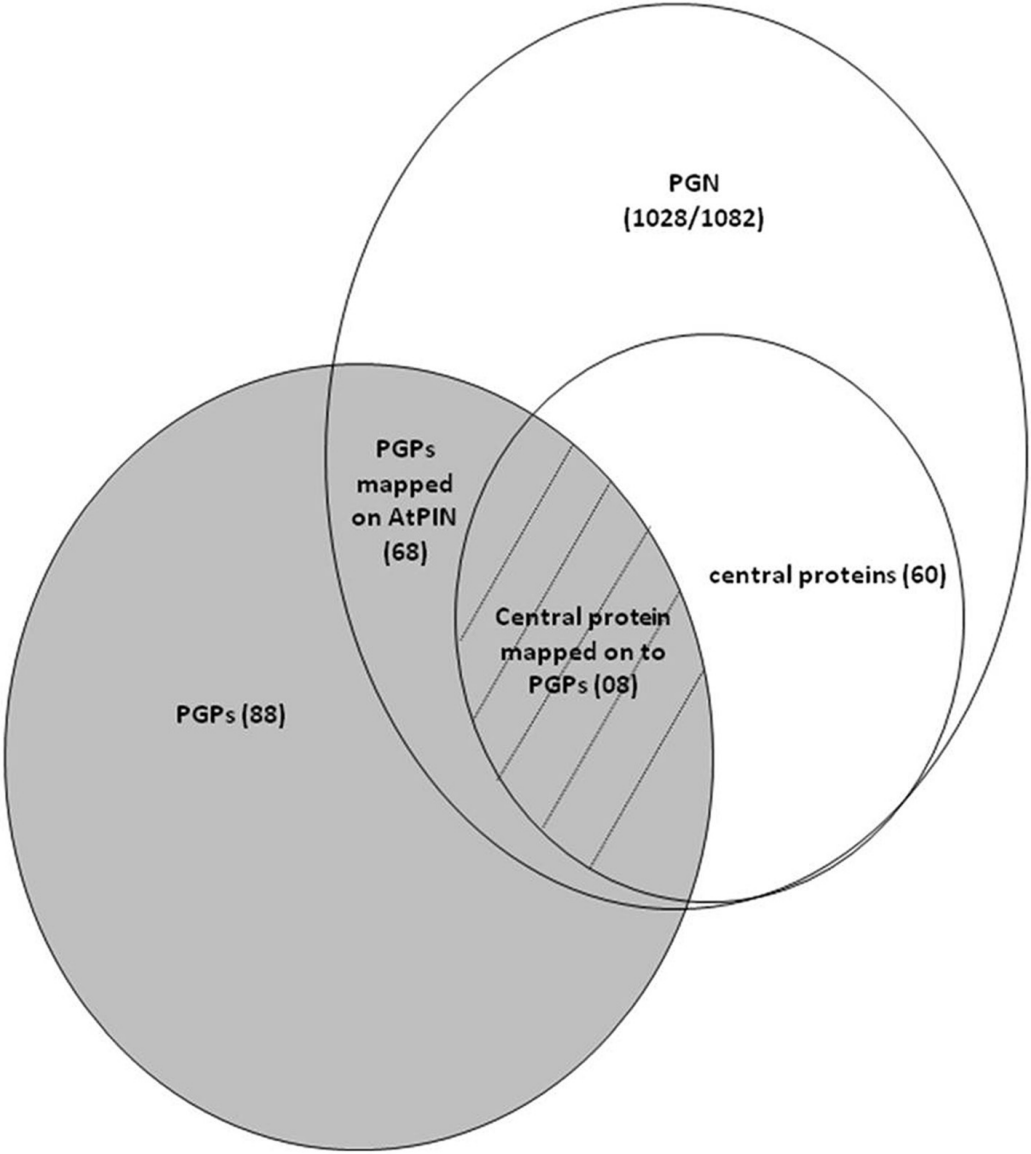
**Venn diagram depicting the strategy used for the identification of germination relevant key proteins from ***Podophyllum*** germination interactome analysis**.

Out of the 60 key proteins that were identified by graph theoretical analysis of PGN, eight protein changes were common to both PGN and differentially accumulated PGPs that were obtained in seed germination protein profile analysis of *Podophyllum hexandrum* (Figure 8) (Dogra et al., 2013). Interestingly, these 8 PGPs are central to the PGN that potentially specifies germination and are also differentially accumulated during radicle protrusion step of seed germination (Figure 2 and Table 2). We looked for the regulatory relevance and role of these proteins in germination mechanisms of *Podophyllum*. It was found that four of these PGPs were up-accumulated and four were down-accumulated (Figure 2). These were broadly associated with signaling, metabolism, protein modification/degradation, cell wall modification and cell cycle regulation. In germinating seeds, soon after rapid water uptake, metabolic processes including hormone signaling, DNA repair, synthesis of proteins from extant and new mRNAs, mobilization and degradation of storage reserves, cell division etc., will take place for completion of germination. Signaling, especially related to phytohormones, plays a decisive role for all these metabolic activities required for the radicle protrusion step of seed germination. In *Podophyllum*, seed coat and thick walled micropylar endosperm are two important constraints for germination which prevent the emergence of expanding embryo (Sreenivasulu et al., 2009). Key proteins identified in PGN also strongly support and validate the earlier findings of protein analysis (Dogra et al., 2013). Thus, proteins involved in the seed coat and endosperm metabolism play regulatory role in promotion of radicle protrusion step of seed germination in *Podophyllum* and possibly in other plant species also.

## Concluding remarks

*Podophyllum* Germination Network (PGN) constructed for the first time using orthologs approach in reference to *Arabidopsis thaliana* truly represents, an extensive interactome underlying potential germination mechanisms in seeds of plants, at least in *Arabidopsis* and *Podophyllum*. Proteome wide information obtained during radicle protrusion step of seed germination by comparing the protein profiles of germinating seeds with that of ungerminated seeds were treated as the critically important proteins for radicle protrusion step of seed germination. As no genomic information is available on *Podophyllum*, data set of protein information obtained in the earlier study (Dogra et al., 2013) was extended for constructing and analyzing the protein interaction network and a system biology tool was utilized by the bioinformatic characterization of potential orthologous proteins of *Arabidopsis*. The data of germination relevant proteins, obtained from proteomic studies, were meaningfully enriched with the proteins that were obtained from systems-level studies of the interactome. The key proteins obtained (88 proteins from proteome analysis and 52 from interactome analysis) might be of critical importance for radicle protrusion step of seed germination in general and that for *Podophyllum* specifically. In the present study, the identified 8 PGPs which are central to the *Podophyllum* protein interaction network also support the “machanosensing” hypothesis of Nonogaki (2014). The proteins thus evidenced may serve as tools for manipulating seed germination in *Podophyllum hexandrum*, and possibly in other plants also, after future line of validation using wet lab experiments. In conclusion, the present study and the other proteomic studies on seed germination of different plant species offers proteins as targets for further characterization of seed germination mechanisms.

## Author contributions

YS conceived the idea and designed the experiments. VD, GB and YS contributed in the acquisition, analysis and interpretation of data and drafting the work in the form of manuscript.

### Conflict of interest statement

The authors declare that the research was conducted in the absence of any commercial or financial relationships that could be construed as a potential conflict of interest.

## Supplementary material

Table S1 **Details of differentially expressed proteins (***Podophyllum*** Germination Proteins; PGPs), identified through proteomic analysis, during seed germination in ***Podophyllum hexandrum*** Royle and their orthologs in ***Arabidopsis thaliana*****.

Table S2 **Details of the predicted pairwise interactions among the ***Arabidopsis*** proteins**.

Table S3 **Nodes with Top50 values identified for each of the parameters; Closeness, Radialilty, Degree and betweenness (stress)**. For Top50 degree-values, 57 such nodes were obtained, whereas 50 nodes each were obtained for the remaining three parameters.

Figure S1 **2DE analysis of proteins of germinating seeds (late Phase II) as compared to un-germinated seeds (early Phase II) during germination**. Marked spots indicate changes in the seed proteome of *Podophyllum* during radicle protrusion step.

Figure S2 *****Arabidopsis*** Protein Interaction Network (***At***PIN) comprises of 96,827 protein interactions among 15,163 proteins (red)**. Orthologs of ***Arabidopsis*** were considered as functional equivalent representatives of PGPs and were mapped onto the ***At***PIN. PGN comprises experimentally identified PGPs and proteins in their functional neighborhood that are reported as their direct interactors (Yellow).

Figure S3 *****Podophyllum*** Germination Network (PGN), comprising of 10,519 interactions among 1082 proteins, was constructed by extending the seed set of 68 (out of 88) ***Podophyllum*** germination proteins, obtained from proteomics studies, to include their interacting partners**. Interactions reported for *Arabidopsis thaliana* (as represented in the *At*PIN) were used as a reference. The giant component comprising 10,466 interactions among 1028 proteins were used for the network analysis.
