# Supplementary material for: Re-analysis of protein data reveals the germination pathway and up accumulation mechanism of cell wall hydrolases during the radicle protrusion step of seed germination in Podophyllum hexandrum- a high altitude plant
Source: Front Plant Sci. 2015 Oct 26;6:874. doi: 10.3389/fpls.2015.00874 (PMC4620410; doi:10.3389/fpls.2015.00874)
Supplement: Table S1 — Details of differentially expressed proteins (Podophyllum Germination Proteins; PGPs), identified through proteomic analysis, during seed germination in Podophyllum hexandrum Royle and their orthologs in Arabidopsis thaliana. [file Table1.DOCX]

**Supplementary Table S1. Details of differentially expressed proteins (*Podophyllum* Germination Proteins; PGPs), identified through proteomic analysis, during seed germination in *Podophyllum hexandrum* Royle and their orthologs in *Arabidopsis thaliana*.**

| Sr. No. | Spot No. | Protein Name | Ortholog in Arabidopsis  (Locus ID) | E Value | Expression during germination |
| --- | --- | --- | --- | --- | --- |
| 1 | 2 | Putative tubulin folding cofactor C | AT4G39920 | 4e-58 | UP |
| 2 | 3 | Putative glycosyltransferase 3 | AT5G07720 | 0.0 | UP |
| 3 | 4 | CRS2-associated factor 2, chloroplastic | AT1G23400 | 0.0 | UP |
| 4 | 6 | Myrosinase 5 | AT1G51470 | 0.0 | UP |
| 5 | 7 | Predicted protein/ Internal NAD(P)H dehydrogenase | AT1G07180 | 0.0 | UP |
| 6 | 8 | AP2-like ethylene-responsive transcription factor TOE2 | AT5G60120 | 0.0 | UP |
| 7 | 9 | Pectinacetylesterase | AT5G23870 | 0.0 | UP |
| 8 | 10 | S-adenosylmethionine synthetase 1 | AT1G02500 | 0.0 | UP |
| 9 | 11 | ATP-dependent Clp protease proteolytic subunit-related protein 1 | AT1G49970 | 0.0 | DN |
| 10 | 12 | Putative cysteine-rich repeat secretory protein 20 | AT3G21933 | 0.0 | DN |
| 11 | 13 | Ent-kaurene synthase B, chloroplast precursor | AT1G79460 | 0.0 | DN |
| 12 | 14 | Putative MtN19 | AT5G61820 | e-119 | UP |
| 13 | 15 | Receptor-like protein kinase HERK 1 | AT3G46290 | 0.0 | DN |
| 14 | 17 | Protein kinase p34cdc2 | AT3G48750 | e-152 | DN |
| 15 | 18 | Short chain dehydrogenase/reductase | AT2G24190 | 0.0 | UP |
| 16 | 19 | Deoxyhypusine synthase | AT5G05920 | 0.0 | UP |
| 17 | 20 | Isopentenyl-diphosphate Delta-isomerase I | AT5G16440 | 0.0 | UP |
| 18 | 21 | Expansin-B8 | AT1G65680 | 0.0 | UP |
| 19 | 22 | Germacrene A synthase short form | AT5G44630 | 1e-118 | UP |
| 20 | 23 | Flavanone 3-hydroxylase | AT3G51240 | 1e-150 | UP |
| 21 | 24 | Probable xyloglucan glycosyltransferase 2 | AT2G06850 | 0.0 | UP |
| 22 | 25 | Probable WRKY transcription factor 65 | AT1G29280 | 0.0 | UP |
| 23 | 26 | Quinone-oxidoreductase homolog | AT4G13010 | 1e-133 | UP |
| 24 | 27 | Putative D-isomer specific 2-hydroxyacid dehydrogenase | AT1G79870 | 2e-96 | UP |
| 25 | 29 | Putative dehydroascorbate reductase | AT5G16710 | 4e-08 | UP |
| 26 | 30 | Adenylate kinase B | AT5G50370 | 1e-109 | UP |
| 27 | 31 | Probable glutathione S-transferase BZ2 | AT2G02390 | 0.0 | UP |
| 28 | 32 | Beta-1,3-endoglucanase | AT3G57240 | 3e-90 | UP |
| 29 | 33 | Maturase K | ATCG00040 | 1e-76 | UP |
| 30 | 34 | Auxin-responsive protein IAA11 | AT4G28640 | 0.0 | UP |
| 31 | 35 | ABA DEFICIENT 2 (ABA2) Xanthoxin dehydrogenase | AT1G52340 | 0.0 | UP |
| 32 | 36 | Kunitz family trypsin and protease inhibitor protein | AT1G17860 | 3e-05 | UP |
| 33 | 37 | Phytochrome N1 | AT5G48150 | 3e-80 | UP |
| 34 | 38 | Light-induced protein (Drought-induced stress protein CDSP-34) | AT1G76080 | 0.41 | UP |
| 35 | 39 | F-box/LRR-repeat protein At3g58900 | AT3G58900 | 0.0 | UP |
| 36 | 40 | E3 ubiquitin-protein ligase makorin | AT3G08505 | 0.0 | UP |
| 37 | 42 | protein kinase family protein (AT3G13690) | AT3G13690 | 0.0 | UP |
| 38 | 43 | Xyloglucan endotransglycosylase | AT5G57560 | 1e-101 | UP |
| 39 | 44 | BTB/POZ domain-containing protein (At4g08455) | AT4G08455 | 0.0 | UP |
| 40 | 45 | GRIK1 (GEMINIVIRUS REP INTERACTING KINASE 1) | AT3G45240 | 0.0 | DN |
| 41 | 46 | 22.7 kDa class IV heat shock protein | AT2G19310 | 0.0 | UP |
| 42 | 47 | TAP42-like family protein | AT5G53000 | 0.0 | DN |
| 43 | 48 | Probable calcium-binding protein CML23 | AT1G66400 | 0.0 | UP |
| 44 | 49 | heat shock protein 60-3A | AT3G13860 | 0.0 | UP |
| 45 | 50 | Hypothetical protein At1g10600 (AMSH-like ubiquitin thiolesterase 2) | AT1G10600 | 0.0 | DN |
| 46 | 51 | 50S ribosomal protein L12 | AT3G27830 | 0.0 | UP |
| 47 | 52 | Chalcone isomerase | AT3G55120 | 0.0 | UP |
| 48 | 53 | Late embryogenesis abundant protein 2 | AT1G02820 | 7e-08 | UP |
| 49 | 56 | GTP-binding nuclear protein Ran-A1 | AT5G20010 | 1e-116 | UP |
| 50 | 59 | myc-like regulatory R protein/ Enhancer of Glabra 3 (MYC) | AT1G63650 | 0.0 | UP |
| 51 | 61 | proteinase inhibitor B | AT2G31980 | 0.002 | UP |
| 52 | 62 | MLP-like protein 423 | AT1G24020 | 0.0 | UP |
| 53 | 63 | Chalcone--flavonone isomerase 2 | AT5G66220 | 0.0 | UP |
| 54 | 64 | Syn-pimara-7,15-diene synthase | AT1G79460 | 1e-173 | DN |
| 55 | 65 | DNA-directed RNA polymerase subunit beta | ATCG00190 | 0.0 | DN |
| 56 | 66 | Glutathione gamma-glutamylcysteinyltransferase 2 | AT1G03980 | 0.0 | DN |
| 57 | 67 | Vicilin C720.0 | AT2G18540 | 1e-51 | DN |
| 58 | 68 | Calcium-dependent protein kinase 3 | AT4G23650 | 0.0 | DN |
| 59 | 69 | Ornithine aminotransferase | AT5G46180 | 2e-90 | DN |
| 60 | 70 | RING1A (RING 1A); protein binding / zinc ion binding | AT5G44280 | 0.0 | DN |
| 61 | 71 | Tubby-like F-box protein 10 | AT1G25280 | 0.0 | DN |
| 62 | 72 | Histone-lysine N-methyltransferase ATX1 | AT2G31650 | 0.0 | DN |
| 63 | 73 | 3-ketoacyl-CoA synthase 16 | AT4G34250 | 0.0 | DN |
| 64 | 74 | 50S ribosomal protein L5 | AT4G01310 | 0.0 | DN |
| 65 | 75 | NAD(P)H-quinone oxidoreductase subunit H | AT3G27890 | 0.0 | DN |
| 66 | 76 | F-box protein At3g13820 | AT3G13820 | 0.0 | DN |
| 67 | 78 | Probable cinnamyl alcohol dehydrogenase | AT1G72680 | 5e-08 | DN |
| 68 | 80 | Putative protease Do-like 14 | AT5G27660 | 0.0 | DN |
| 69 | 81 | 30S ribosomal protein S3 | ATCG00800 | 1e-104 | DN |
| 70 | 82 | Putative protease Do-like 14 | AT5G27660 | 0.0 | DN |
| 71 | 84 | Malate dehydrogenase [NADP] 2 | AT5G58330 | 1e-180 | DN |
| 72 | 85 | DNA-directed RNA polymerase subunit alpha | ATCG00740 | 1e-118 | DN |
| 73 | 86 | F-box protein At4g12560 | AT4G12560 | 0.0 | DN |
| 74 | 87 | Cell division control protein 2 homolog 1 | AT3G54180 | 0.0 | DN |
| 75 | 90 | UPF0496 protein At3g28270 | AT3G28270 | 0.0 | DN |
| 76 | 91 | Xylanase inhibitor protein 1 | AT1G05850 | 0.0 | DN |
| 77 | 93 | Enolase | AT2G29560 | 1e-138 | DN |
| 78 | 94 | transducin family protein / WD-40 repeat family protein | AT1G04140 | 0.0 | DN |
| 79 | 95 | Granule-bound starch synthase 1 | AT1G32900 | 0.0 | DN |
| 80 | 96 | Cyclin-like F-box | AT2G31470 | 3e-09 | DN |
| 81 | 100 | Cyclin-dependent kinase A-2 | AT3G48750 | 1e-145 | DN |
| 82 | 103 | Ribonuclease S-F11 | AT2G02990 | 8e-20 | DN |
| 83 | 105 | DEAD-box ATP-dependent RNA helicase 18 | AT5G05450 | 0.0 | DN |
| 84 | 106 | putative thioredoxin | AT3G02730 | 0.0 | DN |
| 85 | 107 | Non-specific lipid-transfer protein C, cotyledon-specific isoform | AT5G59320 | 6e-11 | DN |
| 86 | 110 | 50S ribosomal protein L18 | AT1G48350 | 3e-38 | DN |
| 87 | 112 | Glutaredoxin-C1 | AT5G63030 | 4e-15 | DN |
| 88 | 114 | Caffeoyl-CoA O-methyltransferase (CCoAMT) | AT4G34050 | e-128 | DN |
