# Supplementary material for: Re-analysis of protein data reveals the germination pathway and up accumulation mechanism of cell wall hydrolases during the radicle protrusion step of seed germination in Podophyllum hexandrum- a high altitude plant
Source: Front Plant Sci. 2015 Oct 26;6:874. doi: 10.3389/fpls.2015.00874 (PMC4620410; doi:10.3389/fpls.2015.00874)
Supplement: Table S3 — Nodes with Top50 values identified for each of the parameters; Closeness, Radialilty, Degree and betweenness (stress). For Top50 degree-values, 57 such nodes were obtained, whereas 50 nodes each were obtained for the remaining three parameters. [file Table3.DOCX]

Table S3: Nodes with Top50 values identified for each of the parameters; Closeness, Radialilty, Degree and betweenness (stress). For Top50 degree-values, 57 such nodes were obtained, whereas 50 nodes each were obtained for the remaining three parameters

| **S. No** | **Closeness** | **Radiality** | **Degree** | **Stress** |
| --- | --- | --- | --- | --- |
| 1 | AT5G52640 | AT5G52640 | AT5G20010 | AT3G03980 |
| 2 | AT3G48750 | AT3G48750 | AT4G27130 | AT5G10450 |
| 3 | AT3G62870 | AT3G62870 | AT4G21490 | AT5G24520 |
| 4 | AT5G09590 | AT5G09590 | AT4G26600 | AT5G12410 |
| 5 | AT1G78770 | AT1G78770 | AT3G22290 | AT1G63650 |
| 6 | AT4G26840 | AT4G26840 | AT2G33800 | AT5G42980 |
| 7 | AT3G45240 | AT3G45240 | AT2G20060 | AT5G36890 |
| 8 | AT5G63400 | AT5G63400 | AT3G22890 | AT2G33210 |
| 9 | AT1G04750 | AT4G37910 | AT2G27970 | AT4G37910 |
| 10 | AT4G37910 | AT1G04750 | AT5G46150 | AT2G18230 |
| 11 | AT3G12280 | AT3G12280 | AT5G09660 | AT3G23990 |
| 12 | AT1G34580 | AT1G34580 | AT4G39200 | AT5G20850 |
| 13 | AT1G71860 | AT1G71860 | AT2G40290 | AT4G02570 |
| 14 | AT4G35620 | AT4G35620 | AT4G33090 | AT5G14320 |
| 15 | AT3G23990 | AT3G23990 | AT3G63490 | AT2G17510 |
| 16 | AT3G18660 | AT3G18660 | AT2G18450 | AT1G66410 |
| 17 | AT3G06720 | AT3G06720 | AT1G47830 | AT3G13860 |
| 18 | AT5G20850 | AT5G20850 | AT1G25260 | AT2G29570 |
| 19 | AT2G31020 | AT2G31020 | AT1G21700 | AT1G20930 |
| 20 | AT2G16740 | AT2G16740 | AT5G54200 | AT3G48750 |
| 21 | AT3G13920 | AT3G13920 | AT1G25155 | AT3G62870 |
| 22 | AT1G02690 | AT1G02690 | ATCG01240 | AT5G52640 |
| 23 | AT2G35390 | AT2G35390 | AT5G01770 | ATCG00800 |
| 24 | AT1G74710 | AT1G74710 | AT4G04695 | AT1G74710 |
| 25 | AT3G12580 | AT3G12580 | AT2G17360 | ATCG00830 |
| 26 | AT5G42190 | AT5G42190 | AT3G48750 | AT2G43030 |
| 27 | AT3G09630 | AT5G06150 | AT3G62870 | AT5G42190 |
| 28 | AT5G06150 | AT3G09630 | AT5G52640 | AT3G43810 |
| 29 | AT4G01370 | AT4G01370 | AT3G54180 | AT3G12280 |
| 30 | AT2G30160 | AT2G30160 | AT3G45240 | AT1G78770 |
| 31 | AT5G10450 | AT5G10450 | AT1G78770 | AT3G54180 |
| 32 | AT3G22890 | AT3G22890 | AT1G04750 | ATCG00160 |
| 33 | AT4G14800 | AT4G14800 | AT2G16740 | AT4G26840 |
| 34 | AT5G19510 | AT5G19510 | AT3G18660 | AT5G63400 |
| 35 | AT2G02760 | AT5G19990 | ATCG00830 | AT1G34580 |
| 36 | AT5G19990 | AT2G02760 | AT3G12280 | AT3G45240 |
| 37 | AT3G54840 | AT3G54840 | AT1G34580 | AT5G09590 |
| 38 | AT4G04700 | AT4G04700 | AT1G07180 | AT3G18660 |
| 39 | AT2G33560 | AT2G33560 | AT5G67380 | AT3G12580 |
| 40 | AT2G18230 | AT2G18230 | AT3G12580 | ATCG00180 |
| 41 | AT1G54270 | AT1G54270 | AT3G09630 | AT1G04750 |
| 42 | AT3G54180 | AT3G54180 | ATCG00800 | AT2G31020 |
| 43 | AT2G43030 | AT2G43030 | ATCG00160 | AT2G16740 |
| 44 | AT1G77670 | AT1G77670 | AT2G43030 | AT5G67380 |
| 45 | AT2G20580 | AT2G20580 | AT4G26840 | AT3G25980 |
| 46 | AT5G67380 | AT5G67380 | AT3G43810 | AT1G07180 |
| 47 | AT4G12620 | AT4G12620 | AT2G31020 | AT4G39330 |
| 48 | AT5G35910 | AT5G35910 | AT5G09590 | AT3G09630 |
| 49 | AT1G60680 | AT4G21490 | AT3G25980 | AT5G21274 |
| 50 | AT4G21490 | AT1G60680 | AT4G04700 | AT4G04700 |
| 51 |  |  | AT1G74710 |  |
| 52 |  |  | AT4G39330 |  |
| 53 |  |  | AT5G42190 |  |
| 54 |  |  | ATCG00180 |  |
| 55 |  |  | AT5G63400 |  |
| 56 |  |  | AT5G21274 |  |
| 57 |  |  | AT5G20010 |  |
