# Supplementary figures and images for: Re-analysis of protein data reveals the germination pathway and up accumulation mechanism of cell wall hydrolases during the radicle protrusion step of seed germination in Podophyllum hexandrum- a high altitude plant
Source: Front Plant Sci. 2015 Oct 26;6:874. doi: 10.3389/fpls.2015.00874 (PMC4620410; doi:10.3389/fpls.2015.00874)

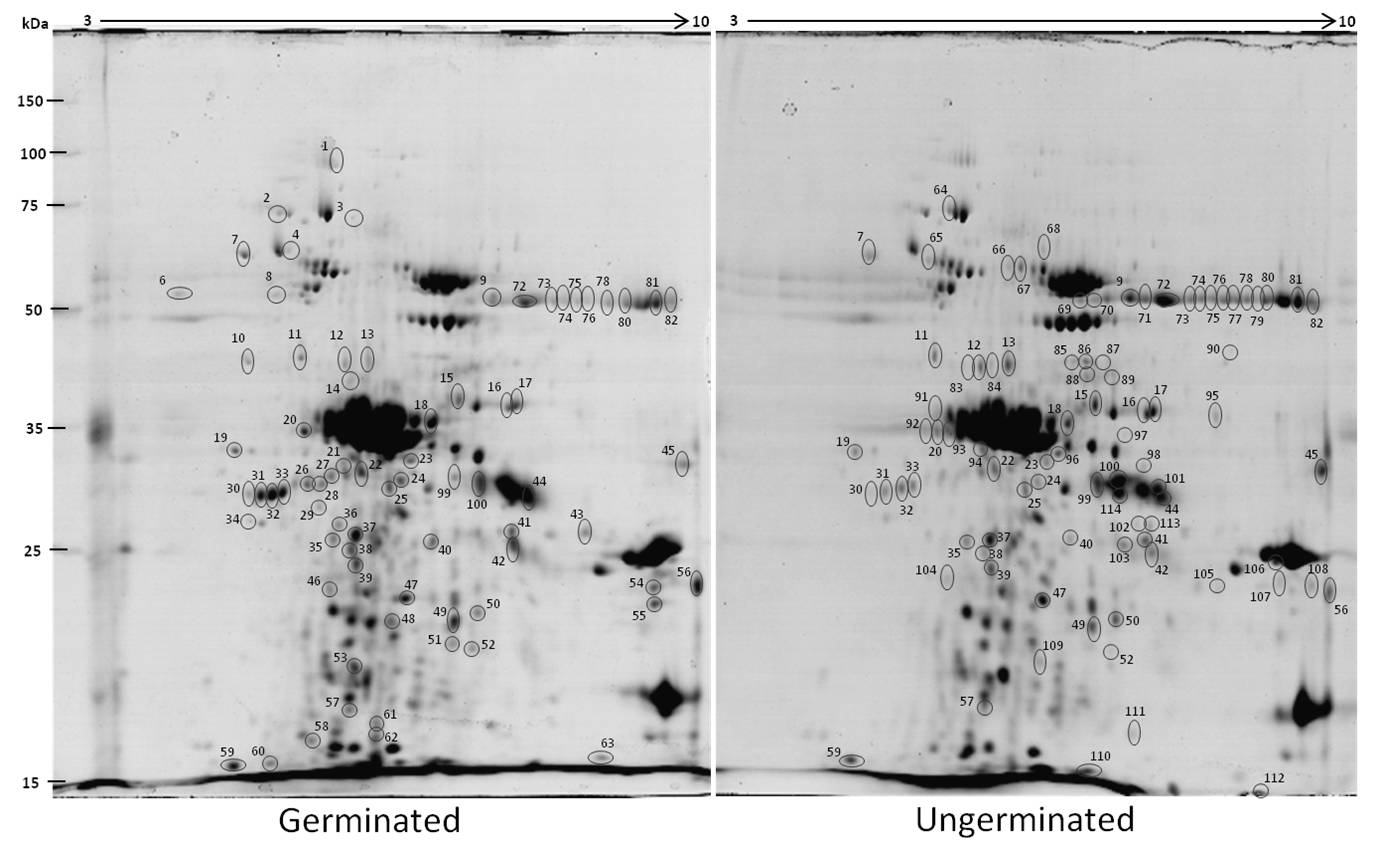

Supplement: Figure S1 — 2DE analysis of proteins of germinating seeds (late Phase II) as compared to un-germinated seeds (early Phase II) during germination. Marked spots indicate changes in the seed proteome of Podophyllum during radicle protrusion step. [file Image1.JPEG]

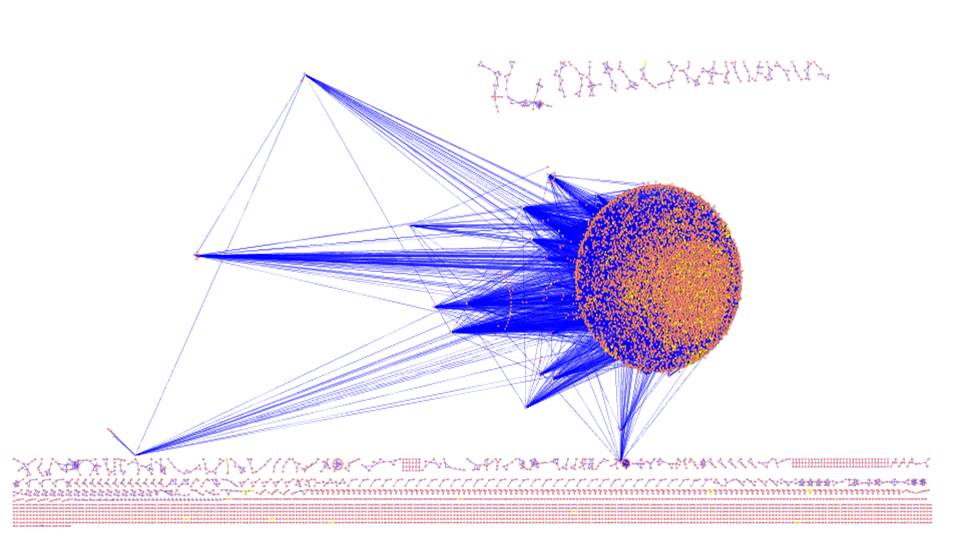

Supplement: Figure S2 — Arabidopsis Protein Interaction Network (AtPIN) comprises of 96,827 protein interactions among 15,163 proteins (red). Orthologs of Arabidopsis were considered as functional equivalent representatives of PGPs and were mapped onto the AtPIN. PGN comprises experimentally identified PGPs and proteins in their functional neighborhood that are reported as their direct interactors (Yellow). [file Image2.JPEG]

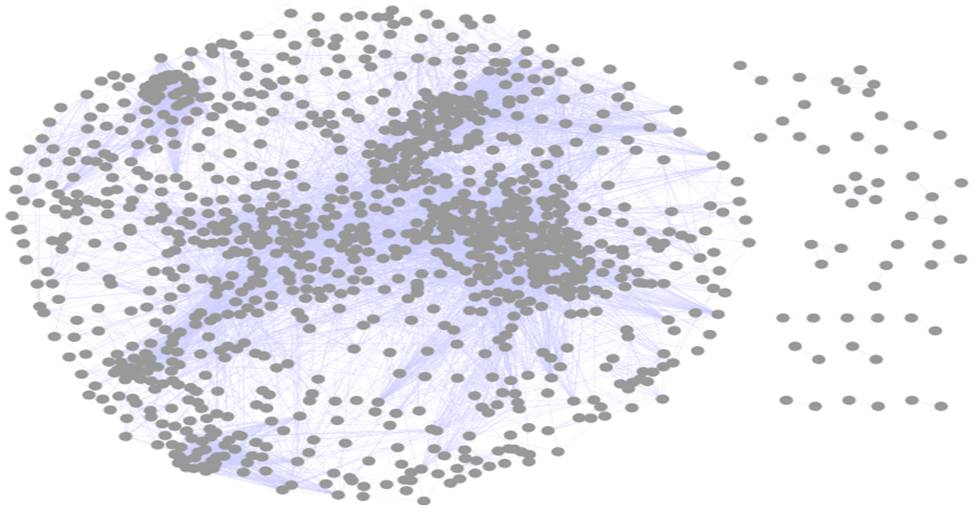

Supplement: Figure S3 — Podophyllum Germination Network (PGN), comprising of 10,519 interactions among 1082 proteins, was constructed by extending the seed set of 68 (out of 88) Podophyllum germination proteins, obtained from proteomics studies, to include their interacting partners. Interactions reported for Arabidopsis thaliana (as represented in the AtPIN) were used as a reference. The giant component comprising 10,466 interactions among 1028 proteins were used for the network analysis. [file Image3.JPEG]
